# Treatment of SARS-CoV-2-induced pneumonia with NAD^+^ and NMN in two mouse models

**DOI:** 10.1038/s41421-022-00409-y

**Published:** 2022-04-29

**Authors:** Yisheng Jiang, Yongqiang Deng, Huanhuan Pang, Tiantian Ma, Qing Ye, Qi Chen, Haiyang Chen, Zeping Hu, Cheng-Feng Qin, Zhiheng Xu

**Affiliations:** 1 State Key Laboratory of Molecular Developmental Biology, CAS Center for Excellence in Brain Science and Intelligence Technology, Institute of Genetics and Developmental Biology, Chinese Academy of Sciences, Beijing, China; 2 University of Chinese Academy of Sciences, Beijing, China; 3 Department of Virology, State Key Laboratory of Pathogen and Biosecurity, Beijing Institute of Microbiology and Epidemiology, Beijing, China; 4 School of Pharmaceutical Sciences, Tsinghua-Peking Center for Life Sciences, Beijing Frontier Research Center for Biological Structure, Tsinghua University, Beijing, China; 5 Research Unit of Discovery and Tracing of Natural Focus Diseases, Chinese Academy of Medical Sciences, Beijing, China; 6 Parkinson’s Disease Center, Beijing Institute for Brain Disorders, Beijing, China

**Keywords:** Mechanisms of disease, Cell death

## Abstract

The global COVID-19 epidemic has spread rapidly around the world and caused the death of more than 5 million people. It is urgent to develop effective strategies to treat COVID-19 patients. Here, we revealed that SARS-CoV-2 infection resulted in the dysregulation of genes associated with NAD^+^ metabolism, immune response, and cell death in mice, similar to that in COVID-19 patients. We therefore investigated the effect of treatment with NAD^+^ and its intermediate (NMN) and found that the pneumonia phenotypes, including excessive inflammatory cell infiltration, hemolysis, and embolization in SARS-CoV-2-infected lungs were significantly rescued. Cell death was suppressed substantially by NAD^+^ and NMN supplementation. More strikingly, NMN supplementation can protect 30% of aged mice infected with the lethal mouse-adapted SARS-CoV-2 from death. Mechanically, we found that NAD^+^ or NMN supplementation partially rescued the disturbed gene expression and metabolism caused by SARS-CoV-2 infection. Thus, our in vivo mouse study supports trials for treating COVID-19 patients by targeting the NAD^+^ pathway.

## Introduction

Novel severe acute respiratory syndrome coronavirus 2 (SARS-CoV-2) has caused the global epidemic of coronavirus disease 2019 (COVID-19). Patients with critical COVID-19 develop severe respiratory distress, and many of them died from it^1^. Although vaccines with high protective potency have been developed, the ability of SARS-CoV-2 to mutate quickly has brought huge uncertainty to the COVID-19 epidemic, which has prioritized the development of drugs for COVID-19 treatment.

Nicotinamide adenine dinucleotide (NAD^+^) and NADH are essential coenzymes for multiple cellular energy metabolism pathways, such as tricarboxylic acid (TCA) cycle and oxidative phosphorylation^2^. NAD^+^ also serves as a substrate for sirtuin deacetylases (SIRTs), cyclic ADP-ribose synthases (including CD38 and CD157), and poly- or mono-(ADP-ribose) polymerases (PARPs), and participate in many important biological processes, including cell survival, aging, inflammation, etc^2–4^. Besides, NAD^+^ can be phosphorylated to nicotinamide adenine dinucleotide phosphate (NADP^+^) by NAD^+^ kinase (NADK)^5^. Both NADP^+^ and its reduced form, NADPH, play critical roles in maintaining redox balance and biosynthesis of fatty acids and nucleic acids^6,7^. In mammalian cells, NAD^+^ is synthesized through the de novo pathway and the salvage pathway^7^.

One of the most striking disturbance in the sera of COVID-19 patients was the increase of metabolites related to de novo production of NAD^+^^8^. Whether and how NAD^+^ metabolism is disrupted in the lungs of COVID-19 patients is still not clear. NAD^+^ has been proposed as a selective inhibitor for SARS-CoV-2 main protease (M^pro^) and multi-functional papain-like protease (PL^pro^)^9,10^. Recently, we found that NAD^+^ supplementation effectively alleviates cell death and inhibits microglia activation in Zika virus (ZIKV)-infected mouse brains^11^. Therefore, it is intriguing to explore whether NAD^+^ or its intermediates are protective from SARS-CoV-2 infection.

In this study, we used transcriptomics and metabolomics of lungs to assess the disturbance of gene expression and metabolism after SARS-CoV-2 infection and tested the protective effect of NAD^+^ and its intermediate, NMN, in lungs in two different mouse models of SARS-CoV-2 infection^12,13^. Numerous gene expression changes and activation of multiple immune pathways in human samples were imitated in our mouse model. SARS-CoV-2-induced pneumonia could be significantly alleviated by NAD^+^ and NMN treatment. We used real-time quantitative PCR (RT-qPCR) to validate our transcriptomics results and uncovered the possible mechanisms by which NAD^+^ and NMN were effective in treating pneumonia caused by SARS-CoV-2 infection. Meanwhile, metabolomics analysis was applied in the lethal mouse model of SARS-CoV-2 infection to reveal the alteration of metabolism after SARS-CoV-2 infection and protective effects of NMN.

## Results

### Transcriptomics reveals similar immune responses in a mouse model of SARS-CoV-2 infection and COVID-19 patients

To explore potential therapeutic targets for COVID-19, we inspected the differentially expressed genes in a mouse-adapted SARS-CoV-2 (MASCp6) infection mouse model^13^. Eight- to nine-month-old (aged) female BALB/c mice were intranasally (i.n.) infected with MASCp6 and sacrificed 3 days post infection (dpi). To confirm the infection of MASCp6 in mouse lungs, SARS-CoV-2 proteins and RNA copies were detected in lung tissues. Immunostaining with SARS-CoV-2 spike protein and nucleocapsid protein antibodies indicated that MASCp6 mainly infected airway epithelial cells and some alveolar epithelial cells near the airways (Supplementary Fig. S1). The infection pattern is similar to another mouse-adapted model of SARS-CoV-2, in which receptor-binding domain in the spike protein was remodeled to facilitate its efficient binding to mouse ACE2^14^.

**Fig. 1:**
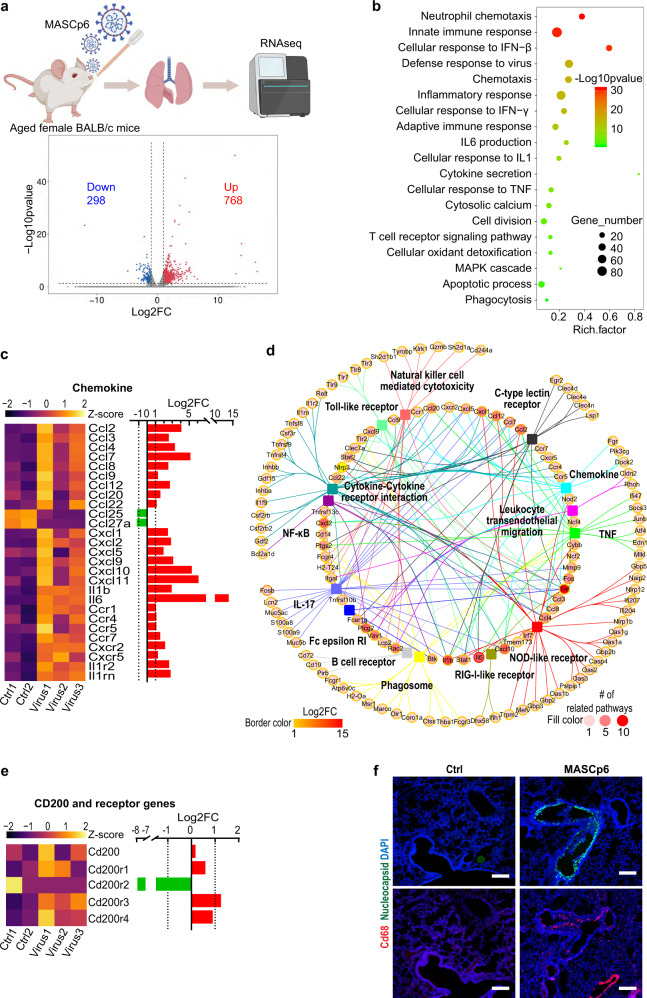
Transcriptomics analysis of MASCp6-infected aged mouse lungs. **a** Overview of the RNA-seq data. Dot plot shows 1066 significantly differentially expressed (*P* < 0.05 and |log_2_FC| > 1) genes with 298 downregulated and 768 upregulated in the infected group. FC, fold change. **b** GO analysis of significantly upregulated genes. Rich factor represents the ratio of significantly differentially expressed genes to the total genes belonging to the GO term. **c** Heatmap and bar plot show relative expression levels of significantly differentially expressed genes of chemokine, interleukin, and their receptors. **d** Network of the genes belonging to significantly-enriched immunity-related KEGG terms of significantly upregulated genes. **e** Heatmap and bar plot show relative expression levels of CD200 and its receptor genes. **f** Immunostaining results with SARS-CoV-2 nucleocapsid (green) and macrophage marker (CD68, red) antibodies of lung sections from MASCp6-infected and control aged mice. Scale bars, 200 μm.

Transcriptomics analysis identified a total of 1066 significantly differentially expressed genes (|log_2_FoldChange (FC)| > 1, *P* < 0.05) with 298 downregulated and 768 upregulated genes in the infected lungs (Fig. 1a). Gene ontology (GO) enrichment analysis of significantly upregulated genes revealed the strong activation of immune-related biological processes, including neutrophil chemotaxis, innate immune response, adaptive immune response, cellular response to IFN, TNF, and IL1, as well as apoptotic process and phagocytosis (Fig. 1b). Infection led to robust induction of chemokine and interleukin genes, including *Il1b*, *Il6*, *Ccl2*, *Ccl3*, *Ccl4*, *Ccl7*, *Cxcl1*, *Cxcl2*, *Cxcl5*, *Cxcl9*, and *Cxcl10*, and related receptor genes, including *Ccr1*, *Ccr4*, *Ccr5*, *Ccr5*, *Ccr7*, *Cxcr2*, *Cxcr5*, *Il1r2*, and *Il1rn* (Fig. 1c). Many significantly upregulated genes participated in neutrophil chemotaxis, including *S100a8* and *S100a9* (Fig. 1b; Supplementary Fig. S2a). These results indicated that innate immune response is activated robustly as that in COVID-19 patients^15–17^.

To inspect the induced immune-related genes and pathways by MASCp6 in more detail, we performed KEGG enrichment analysis. Cytokine–cytokine receptor interaction, NOD-like receptor signaling, IL-17 signaling, chemokine signaling, TNF signaling, Toll-like receptor signaling, as well as other immune pathways were detected (Fig. 1d). Among all significantly upregulated genes in these pathways, *Tnf*, *Il1b*, *Il6*, *Cxcl2*, *Cxcl10*, and *Plcg2* play roles in multiple signaling pathways, suggesting their importance and potential drug targets in SARS-CoV-2 infection (Fig. 1d). In addition, marker genes of neutrophil, macrophage, dendritic cell, B cell, and CD4^+^ T cell were upregulated in infected mice as that in COVID-19 patients (Supplementary Fig. S2c)^18^. Moreover, we found two CD200 receptor genes that are responsible for transmitting strong activating signals^19,20^, *Cd200r3* and *Cd200r4*, were upregulated. This was consistent with the immunofluorescence results showing that substantially more activated macrophages (CD68^+^) were detected in the infected lung section compared to control (Fig. 1f).

Interestingly, we found that 30.2% (90/298) significantly downregulated genes are long non-coding RNAs (lncRNAs) (Supplementary Fig. S3a). This suggests that lncRNAs may play an important role in SARS-CoV-2-induced immune response, based on the fact that lncRNAs have the capacity to regulate the expression of proinflammatory genes^21^.

The above results indicated that SARS-CoV-2-infected murine model mimics some of the immune features present in patients^15,18,22,23^, and it is suitable for testing drugs.

### Cell death occurs with the development of immune response in the SARS-CoV-2-infected mice

**Fig. 2:**
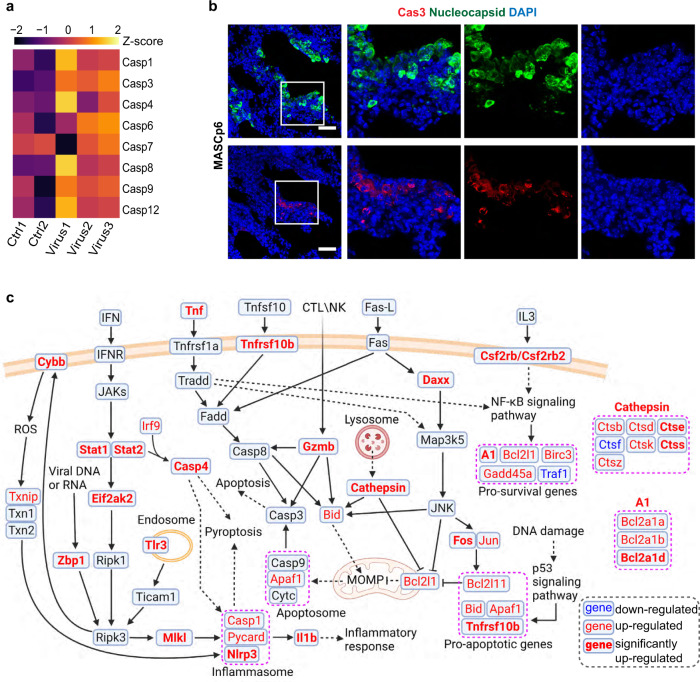
MASCp6 infection leads to cell death in the lungs. **a** Heatmap shows relative expression levels of caspase family genes in the RNA-seq data. **b** Immunostaining results with SARS-CoV-2 nucleocapsid (green) and activated form of caspase 3 (Cas3, red) antibodies of lung sections from MASCp6-infected and control aged mice. Scale bars, 50 μm. **c** Schematic diagram (created in BioRender.com) of apoptosis and necroptosis pathways. MOMP, mitochondrial outer membrane permeabilization. Downregulated: *P* < 0.05 and FC < 1; upregulated: *P* < 0.05 and FC > 1; significantly upregulated: *P* < 0.05 and FC > 2.

SARS-CoV-2 infection induces high expression of massive inflammatory genes and even triggers the cytokine storm, which is considered to be the main cause of death in severe COVID-19 patients^24^. Meanwhile, induction of inflammatory genes in MASCp6-infected mouse lungs might affect cell survival as many of them play roles in the cell death pathways (Supplementary Fig. S2b). In addition, many caspase family members were upregulated after infection (Fig. 2a). The presence of many cleaved caspase 3^+^ cells confirmed that apoptosis occurred in infected lungs (Fig. 2b).

We summarized the apoptosis and necroptosis pathways combining with our transcriptomics data (Fig. 2c). In the apoptosis pathway, *Daxx* and *Fos* were significantly upregulated, which would induce the expression of pro-apoptotic gene, *Bcl2l11* (*Bim*). Other pro-apoptotic genes, *Bid*, *Apaf1,* and *Tnfrsf10b* were induced in the p53 signaling pathway. In addition, the significant upregulation of *Gzmb* and cathepsin family genes, *Ctss* and *Ctse*, suggested the occurrence of mitochondrial outer membrane permeabilization and apoptosome formation^25^. In the JAK-STAT signaling pathway, *Stat1*, *Stat2*, and their target gene, *Casp4*, and inflammasome member, *Nlrp3*, were significantly induced. As the sensor component of inflammasome, NLRP3 plays a crucial role in innate immunity and inflammation in response to viral RNA^26,27^. CASP4 acts as essential effectors of NLRP3 inflammasome-dependent CASP1 activation and IL1B secretion. Both CASP1 and CASP4 can initiate and promote pyroptosis through GSDMD cleavage and activation^28^. Moreover, a group of pro-survival NF-κB signaling pathway downstream genes, *Bcl2a1a*, *Bcl2a1b*, *Bcl2a1d*, *Bcl-XL* (*Bcl2l1*), *Birc3*, and *Gadd45a* were also induced, implicating the compensation or rescue response by surviving cells. Thus, cell death or survival depends on the balanced effects of these genes.

### Elevated expression of NADPH oxidase genes and PARPs dysregulates NAD system in SARS-CoV-2-infected mice

**Fig. 3:**
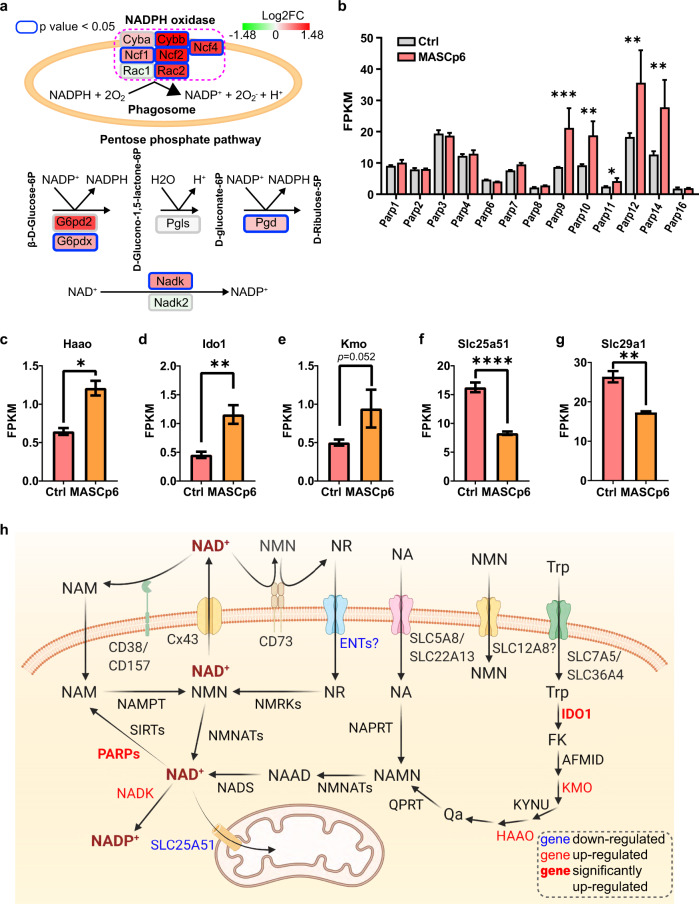
MASCp6 infection leads dysregulation of NAD(H) and NADP(H) related genes. **a** Schematic diagram of NADPH oxidase complex and oxidative phase of pentose phosphate pathway. The fill color of gene represents relative expression level in lungs of MASCp6-infected and control mice. **b** Bar plot shows relative expression levels of PARPs. **c**–**g** Bar plots show relative expression levels of three NAD^+^ de novo synthesis-related genes, *Haao* (**c**), *Ido1* (**d**), and *Kmo* (**e**), and two nucleoside transporter genes, *Slc25a51* (**f**) and *Slc29a1* (**g**). **h** Schematic diagram (created in BioRender.com) of NAD^+^ metabolism pathway (referred to ref. ^3^). Downregulated: *P* < 0.05 and FC < 1; upregulated: *P* < 0.05 and FC > 1; significantly upregulated: *P* < 0.05 and FC > 2. NA, nicotinic acid; NAMN, nicotinic acid mononucleotide; NAAD, nicotinic acid adenine dinucleotide; NR, nicotinamide riboside; NAM, nicotinamide; NMN, nicotinamide mononuclotide; Trp, tryptophan; FK, formyl-kynurenine; Qa, quinolinate. All gene expression data are from RNA-seq. All data are shown as means ± SEM. *P < 0.05, **P < 0.01, ***P < 0.001, ****P < 0.0001.

We noticed that *Cybb*, the critical component of NADPH oxidase that generates reactive oxygen species (ROS), was significantly induced in MASCp6-infected mouse lungs (Fig. 2c). NADPH oxidase plays an important role in neutrophil extracellular trap formation and phagosome maturation during immune response^29^. Intriguingly, other NADPH oxidase component genes, *Ncf1*, *Ncf2*, *Ncf4*, and *Rac2* were also significantly upregulated, suggesting that more NADPH would be consumed to produce ROS (Fig. 3a).

The major source of NADPH in animals is the oxidative phase of pentose phosphate pathway, in which NADPH is produced from NADP^+^ by glucose-6-phosphate dehydrogenase (G6PDH) and 6-phosphogluconate dehydrogenase (PGD)^30^, while NADP^+^ is synthesized from NAD^+^ by NADK^5^ (Fig. 3a). The induction of *G6pd2*, *G6pdx*, *Pgd,* and *Nadk* implicated that more NADPH was produced to respond to the increased demand for NADPH in the immune process after MASCp6 infection.

NAD^+^ is essential for multiple metabolic pathways^3^. We recently found that PARPs, one of main NAD^+^-responsive signaling family, were significantly upregulated while NAD^+^ levels were substantially reduced in ZIKV-infected mouse brains^11^. In MASCp6-infected lungs, *Parp9*, *Parp10*, and *Parp14*, the three PARP members which regulate immune and inflammatory responses^3,31^, were also significantly induced (Fig. 3b). We therefore explored the expression of other genes involved in NAD^+^ biosynthetic process and found that some of those required for NAD^+^ de novo synthesis such as *Haao*, *Ido1*, and *Kmo*, were upregulated (Fig. 3c–e; Supplementary Fig. S4a). This was consistent with the finding in COVID-19 patients^8^. In addition, the downregulation of mitochondrial NAD^+^ transporter gene, *Slc25a51*, and a nucleoside transporter gene, *Slc29a1* (*Ent1*), suggested that both the import of NAD^+^ from the cytoplasm into the mitochondria and the import of nicotinamide riboside (NR) into cells were hindered (Fig. 3f, g)^32–35^. Loss of SLC25A51 has been shown to lead to impaired mitochondrial functions^34^, and a group of mitochondria-encoded genes, including *mt-Atp6*, *mt-Atp8*, *mt-Co1-3*, *mt-Cytb*, and *mt-Nd1-6* were downregulated^36^, although the expressions of genes associated with the TCA cycle and oxidative phosphorylation were not considerably altered in general (Supplementary Fig. S4b, c). Together with the evidence of significantly upregulated expression of *Gzmb* and cathepsin family genes, mitochondrial functions can be predicated to be affected in the infected lungs.

The above results suggest that over-consumption of NADPH by upregulation of NADPH oxidase genes would lead to the convert of more NAD^+^ to NADP^+^, and NADP^+^ is transformed into NADPH by G6PD and PGD in the pentose phosphate pathway in the infected lungs. On the other hand, upregulation of PARPs would also lead to reduced NAD^+^ level. The reduction of NAD^+^ level would stimulate the compensatory de novo synthesis of NAD^+^ (Fig. 3h).

### NAD^+^ supplementation alleviates SARS-CoV-2-induced pneumonia

**Fig. 4:**
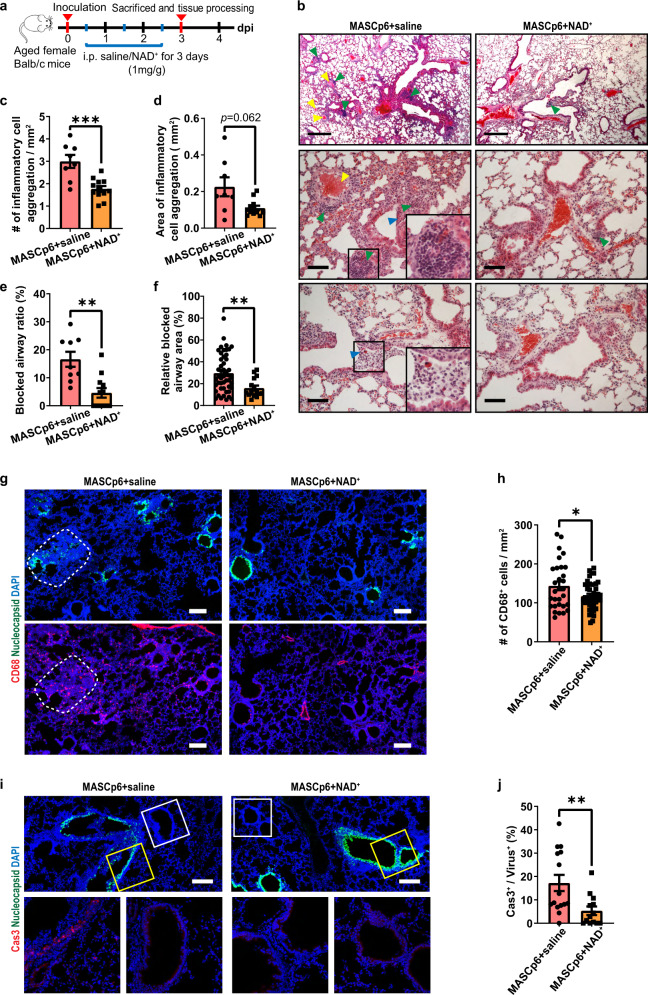
NAD^+^ supplementation alleviates pathological phenotypes of MASCp6-induced pneumonia. **a** Schematic diagram of experimental design for NAD^+^ supplementation in MASCp6-infected aged mice. **b** Hematoxylin and eosin (H&E) staining results show that cell infiltration (green arrowhead), airway blockage (blue arrowhead) and hemolysis (yellow arrowhead) were rescued by NAD^+^ supplementation in lungs of MASCp6-infected aged mice. Scale bars, 500 μm (top panels) and 50 μm (middle and bottom panels). **c**–**f** Quantification of the density (**c**) and area (**d**) of inflammatory cell aggregates and the ratio (**e**) and relative area (**f**) of blocked airway. *t*-test (**c**, **d**) and Mann–Whitney test (**e**, **f**). **g** Immunostaining results with SARS-CoV-2 nucleocapsid (green) and macrophage marker (CD68, red) antibodies of two adjacent lung sections for each group. White border: excessive aggregation of macrophages. Scale bars, 200 μm. **h** Quantification of the density of CD68^+^ cells of saline- and NAD^+^-administered MASCp6-infected aged mice. *t*-test. **i** Immunostaining results with SARS-CoV-2 nucleocapsid (green) and activated form of caspase 3 (Cas3, red) antibodies of two adjacent lung sections for each group. Yellow border: infected airway epithelial cells; White border: airway epithelial cells without virus. Scale bars, 200 μm. **j** Quantification of the ratio of Cas3^+^ cells to infected cells of saline- and NAD^+^-administered MASCp6-infected aged mice. Mann–Whitney test. All quantification data are shown as means ± SEM. *P < 0.05, **P < 0.01, ***P < 0.001, ****P < 0.0001. *n* = 3 mice for MASCp6 + saline group, *n* = 4 mice for MASCp6 + NAD^+^ group.

Based on the observations that SARS-CoV-2 infection led to differential expression of NAD^+^-associated genes in both mouse and human sera (our study and ref. ^37^), we investigated whether NAD^+^ supplementation could rescue MASCp6-induced pneumonia. We treated aged (8–9 months old) and young (6–7 weeks old) infected female mice with NAD^+^ (1 mg/g/day intraperitoneally (i.p.) for 3 days), and sacrificed them on 3 dpi (Fig. 4a).

For aged mice, pathological features including severe inflammatory cell infiltration, alveolar septal thickening, hemolysis, epithelial damage, airway blockage, and cell death are detected in MASCp6-infected lungs (saline group), similar to that in the hACE2 mouse model (Fig. 4b)^38,39^. Compared with the saline group, the number and size of inflammatory cell aggregates as well as the ratio and relative area of blocked airway were substantially reduced in the NAD^+^ treatment group (Fig. 4b–f; Supplementary Fig. S5a). Notably, no hemolysis was detected after treatment with NAD^+^ (Fig. 4b). For young mice, the pathological features were mild, and eliminated almost entirely by NAD^+^ treatment (Supplementary Fig. S6b, c). However, comparable levels of viral mRNA as measured by qPCR and fluorescence intensity as measured by immunostaining with the nucleocapsid antibody were detected in saline and NAD^+^ treatment groups (Supplementary Figs. S5b–d, S6a). This indicates that the treatment relieves the pathological damages, but not viral replication.

Previous studies have shown that SARS-CoV-2 infection induces the infiltration of neutrophils and macrophages in lungs of patients^38,40^. We found that the number of macrophages was reduced significantly in NAD^+^-treated aged and young mice (Fig. 4g, h; Supplementary Fig. S6d, e). Meanwhile, NAD^+^ treatment remarkably suppressed cell death as shown by the reduction of cleaved caspase 3^+^ cells (68.9%) in aged mice (Fig. 4i, j).

The above results indicate that NAD^+^ supplementation can protect the lung from inflammatory injury, including cell death, caused by SARS-Cov-2 infection in both aged and young mice.

### NAD^+^ supplementation partially salvages the changes in gene expression caused by SARS-CoV-2 infection

**Fig. 5:**
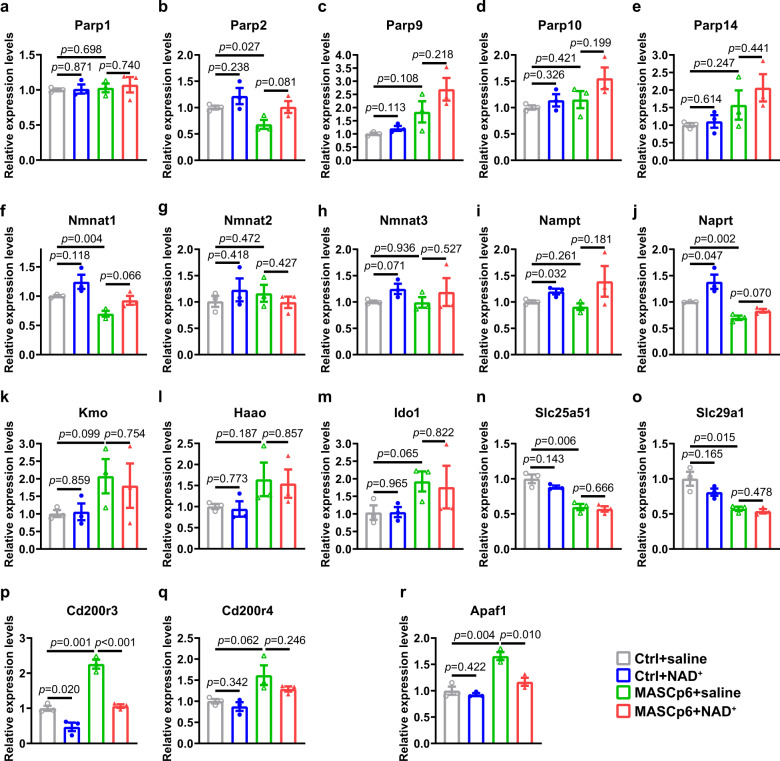
Dysregulation of genes can be partially rescued by NAD^+^ supplementation. **a**–**e** qPCR results of relative expression levels of PARP family members as listed. *Parp1* (**a**), *Parp2* (**b**), *Parp9* (**c**), *Parp10* (**d**), and *Parp14* (**e**). **f**–**j** qPCR results of relative expression levels of NAD^+^ salvage pathway-related genes, *Nmnat1* (**f**), *Nmnat2* (**g**), *Nmnat3* (**h**), *Nampt* (**i**), and *Naprt* (**j**). **k**–**o** qPCR results of relative expression levels of three NAD^+^ de novo synthesis-related genes, *Haao* (**k**), *Ido1* (**l**), and *Kmo* (**m**), and two nucleoside transporter genes, *Slc25a51* (**n**) and *Slc29a1* (**o**). **p**, **q** qPCR results of relative expression levels of CD400 receptor genes, *Cd200r3* (**p**) and *Cd200r4* (**q**). **r** qPCR results of relative expression levels of apoptosis protease activating factor gene, *Apaf1*. All quantification data are shown as means ± SEM, *t*-test. *n* = 3 mice for each group. Exact *P* values are indicated.

To validate the results of RNA-seq and examine whether the changes in gene expression could be rescued by NAD^+^ supplementation, we performed qPCR to inspect the expression of PARP family genes in the infected lungs (Fig. 5a–e). We found that the reduction of *Parp2* expression was rescued almost completely (Fig. 5b). Unexpectedly, the upregulations of *Parp9*, *Parp10,* and *Parp14* were induced further in the NAD^+^ treatment group (Fig. 5c–e). This suggests that *Parp9*, *Parp10*, and *Parp14* are important for host cells to defend against SARS-CoV-2. Host cells had to downregulate the expression of *Parp2* to meet the need of NAD^+^ which was enhanced by elevated expression of *Parp9*, *Parp10*, and *Parp14*.

We also looked at the effect of NAD^+^ supplementation on the expression of genes associated with NAD^+^ synthesis (Fig. 5f–m). Interestingly, the downregulation of genes responsible for NAD^+^ salvage pathway, especially *Nmnat1* and *Naprt*, were partially rescued by NAD^+^ treatment (Fig. 5f, j), but the genes responsible for NAD^+^ de novo pathway were not affected (Fig. 5k–m). In addition, NAD^+^ treatment had no effect on the expression of *Slc25a51* and *Slc29a1* (Fig. 5n, o).

The upregulation of *Cd200r3* was rescued completely while the expression of *Cd200r4* was partially suppressed by NAD^+^ treatment (Fig. 5p, q). These results were matched with reduction of CD68^+^ cells in the lungs (Supplementary Fig. S5e). This indicates that NAD^+^ treatment leads to reduced activation of macrophage. Moreover, the upregulation of *Apaf1*, the apoptosis protease activating factor, was also rescued very significantly by NAD^+^ treatment (Fig. 5r).

Taken together, our qPCR results confirmed many of our findings from RNA-seq analysis and NAD^+^ supplementation was beneficial to the maintenance of the NAD system. In addition, severe inflammatory cell infiltration and cell death caused by SARS-CoV-2 infection might be rescued by NAD^+^ treatment through suppressing the expression of *Cd200r3*, *Cd200r4*, and *Apaf1*, and other related genes untested.

### NMN supplementation alleviates SARS-CoV-2-induced pneumonia

**Fig. 6:**
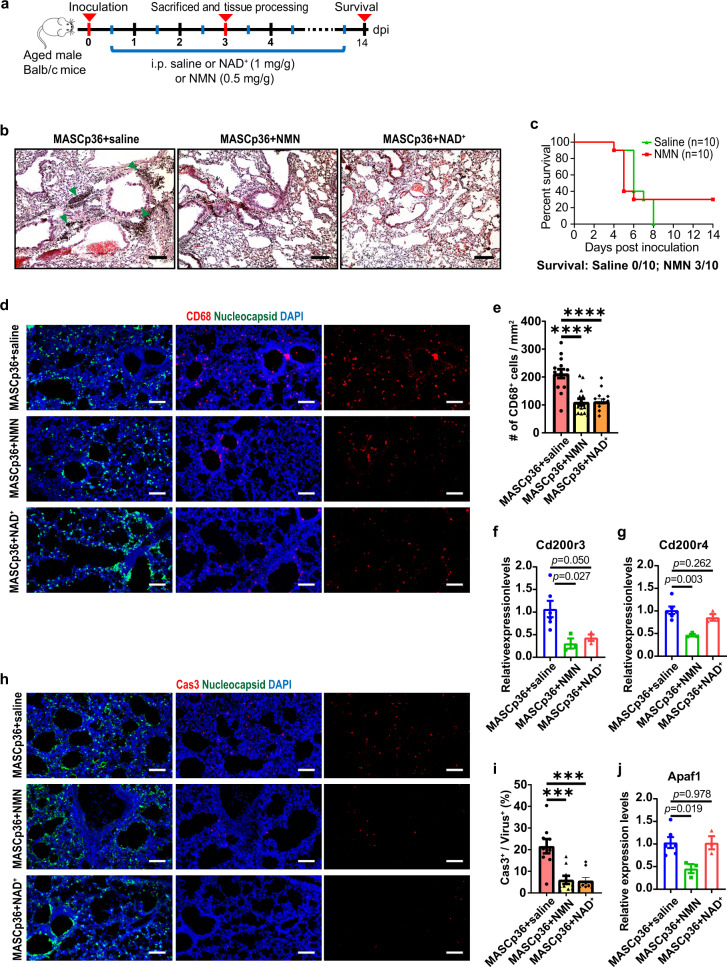
NMN supplementation alleviates pathological phenotypes of MASCp36-induced pneumonia. **a** Schematic diagram of experimental design for NMN and NAD^+^ supplementation in MASCp36-infected aged mice. **b** H&E staining results of lung sections from saline-, NMN-, and NAD^+^-administered MASCp36-infected aged mice. Green arrowhead: inflammatory cell aggregates. Scale bars, 100 μm. **c** Survival of saline (*n* = 10) and NMN (*n* = 10) administered MASCp36-infected aged mice. **d** Immunostaining results with SARS-CoV-2 nucleocapsid (green) and macrophage marker (CD68, red) antibodies of two adjacent lung sections for each group. Scale bars, 100 μm. **e** Quantification of the density of CD68^+^ cells of lung sections from saline-, NMN-, and NAD^+^-administered MASCp36-infected aged mice. *t*-test. *n* = 3 mice per group. **f**, **g** qPCR results of relative expression levels of CD400 receptor genes, *Cd200r3* (**f**) and *Cd200r4* (**g**). *t*-test. *n* = 6 mice for saline group and *n* = 3 mice for NMN and NAD^+^ groups. Exact *P* values are indicated. **h** Immunostaining results with SARS-CoV-2 nucleocapsid (green) and Cas3 (red) antibodies of two adjacent lung sections for each group. Scale bars, 100 μm. **i** Quantification of the ratio of Cas3^+^ cells to infected cells of saline-, NMN-, and NAD^+^-administered MASCp36-infected aged mice. Mann–Whitney test. *n* = 3 mice per group. **j** qPCR results of relative expression levels of apoptosis protease activating factor gene, *Apaf1*. *t*-test. *n* = 6 mice for saline group and *n* = 3 mice for NMN and NAD^+^ groups. Exact *P* values are indicated. All quantification data are shown as means ± SEM. ***P < 0.001, ****P < 0.0001.

To examine whether NAD^+^ intermediates have similar therapeutic effects on SARS-CoV-2-induced pneumonia, we adopted a recently developed and more severe infection model in which mice were infected with a mouse-adapted SARS-CoV-2, MASCp36^12^. Infected mice (8–9 months old) were treated with NMN or NAD^+^ (Fig. 6a). The dosage of NMN (500 mg/kg/day) was widely used in mouse models^41^, which is equal to 55 mg/kg/day in humans according to body surface area^42^. Compared with the severe inflammatory cell infiltration and alveolar septal thickening detected in the saline group, the pathological damage was alleviated obviously in both NMN and NAD^+^ groups (Fig. 6b). More strikingly, 30% of the mice survived eventually in the NMN group, while 100% of the mice died on 8 dpi in the saline group (Fig. 6c). In addition, both the number of CD68^+^ cells and the expression of *Cd200r3* were reduced significantly in both NMN and NAD^+^ groups (Fig. 6d–f). Moreover, cell death was substantially suppressed by NMN and NAD^+^ treatment (72.0% and 73.9%, respectively) (Fig. 6h, i). The suppression of *Cd200r4* and *Apaf1* expression was significant in the NMN group, but not in NAD^+^ group of MASCp36-infected lungs (Fig. 6g, j).

These results indicate that NMN supplementation can suppress the expression of *Cd200r3*, *Cd200r4*, and *Apaf1* in the lethal mouse-adapted SARS-CoV-2 mouse model, and alleviate inflammatory injury in the lungs and even death of the infected animals.

### NMN supplementation partially rescues the disturbance of metabolism caused by SARS-CoV-2 infection

**Fig. 7:**
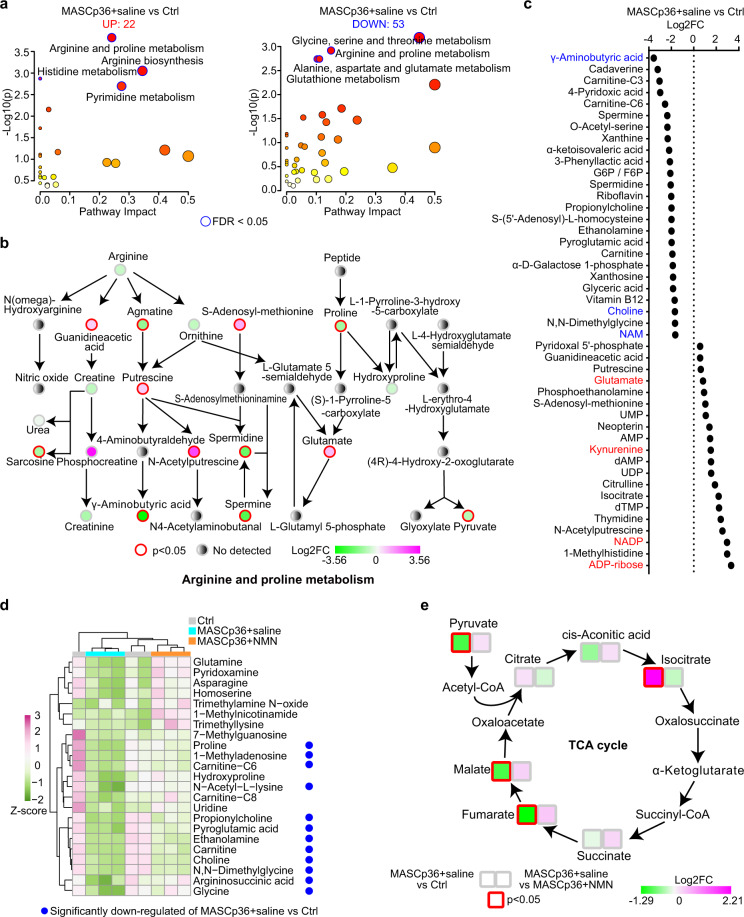
Metabolomics analysis of MASCp36-infected aged mouse lungs. **a** KEGG analysis of significantly differential metabolites (*P* < 0.05; FC < 0.8 or FC > 1.25) between the lungs of saline group of MASCp36-infected mice and control mice. **b** Schematic diagram depicting the dysregulation of arginine and proline metabolism pathways in response to MASCp36 infection. **c** Dot plot shows the top 25 most upregulated and top 20 most downregulated metabolites. **d** Heatmap shows relative abundance of significantly differential metabolites in the NMN group compared with saline group of MASCp36-infected mice. The blue dots mean that the metabolite was significantly downregulated in saline group of MASCp36-infected mice compared with control mice. **e** Schematic diagram depicting that dysregulation of metabolites belonging to TCA cycle in MASCp36-infected mice was partially rescued by NMN supplementation. Nodes without color-block represent metabolites which were not tested.

We next investigated in more detail the protective effect of NMN by targeted metabolomics analysis (Supplementary Table S1). We compared the metabolomes between the MASCp36-infected group (MASCp36 + saline) and the control group (uninfected mice of the same sex and age). A total of 75 significantly deregulated metabolites with 22 upregulated (*P* < 0.05 and fold change > 1.25) and 53 downregulated (*P* < 0.05 and fold change < 0.8) were detected in infected group (Fig. 7a; Supplementary Fig. S7b). We performed KEGG pathway enrichment analysis and found that the arginine and proline metabolism, glycine, serine and threonine metabolism, alanine, aspartate and glutamate metabolism, arginine biosynthesis, glutathione metabolism, histidine metabolism, and pyrimidine metabolism were significantly affected by MASCp36 infeciton (FDR < 0.05) (Fig. 7a, b; Supplementary Fig. S7c). Unexpectedly, the downregulation of γ-aminobutyric acid (GABA) was most significant one in the lungs after MASCp36 infection (Fig. 7b, c). Intracortical GABAergic dysfunction has been postulated as the cause of seizure in COVID-19 patients^43,44^, and administration of GABA or GABAa receptor agonists could limit pneumonitis and death in coronavirus-infected mice^45^. Therefore, GABA is a potential candidate to treat SARS-CoV-2 infection. The significant upregulation of NADP^+^ was consistent with our transcriptomics analyses, in which *Nadk* was significantly upregulated (Figs. 3a, 7c), and ADP-ribose was the most significantly upregulated one in the lungs after MASCp36 infection (Fig. 7c). It is possible that the first macrodomain of SARS-CoV-2 nonstructural protein 3 (Nsp3), Mac1, which has de-mono-ADP-ribosylation activity, counters ADP-ribosylation by PARPs^46^. In addition, the downregulation of choline and upregulation of kynurenine and glutamate in the sera have been reported to be associated with the severity of COVID-19 patients (Fig. 7c)^47–50^.

We then compared the metabolomes between the saline and the NMN groups of MASCp36-infected mice. A total of 23 significantly differential metabolites were detected in the NMN group, and all of them were upregulated (*P* < 0.05 and fold change > 1.25), including glutamine, asparagine, proline, choline, and glycine (Fig. 7d). These metabolites participate in the aminoacyl-tRNA biosynthesis, alanine, aspartate and glutamate metabolism, glycine, serine and threonine metabolism and arginine biosynthesis (Supplementary Fig. S7d). Metabolites and proteins in citrate cycle (TCA cycle) were altered in the sera of COVID-19 patients and in vitro SARS-CoV-2 infection model, respectively^48,50^. We observed that the levels of pyruvate, fumarate and malate were significantly reduced, but isocitrate was significantly increased by MASCp36 infection (Fig. 7e). NMN supplementation could rescue the alterations in TCA cycle metabolites overall (Fig. 7e). One of major dysregulated metabolic pathways of COVID-19 was the tryptophan and kynurenine pathway. Lower level of tryptophan and higher level of kynurenine were found in the sera of severe COVID-19 patients^51^. Intriguingly, we found that the levels of both tryptophan and kynurenine were increased by NMN supplementation in MASCp36-infected mice (Supplementary Fig. S7e). The elevation of glucose in the plasma was considered as an indicator for the severity of COVID-19 patients^47,48^. Our results showed the level of glucose had a tendency of increase in the lungs of MASCp36-infected mice, and the level returned to normal in two NMN supplementation mice (Supplementary Fig. S7e). The reduction of GABA was partially rescued, which also helped to limit pneumonitis and seizure (Supplementary Fig. S7e)^45^. Furthermore, we performed the absolute quantifications of NAD^+^ and its precursors (Supplementary Table S2). Our results indicated that NAD^+^, NMN, NR, and NAM were elevated in the lungs of MASCp36-infected mice after NMN supplementation (Supplementary Fig. S7f).

Our targeted metabolomics analyses indicate that the disturbed metabolism in the sera of COVID-19 patients was partly reproduced in the lungs of our mouse model, and it could be ameliorated by NMN supplementation.

## Discussion

In this study, our transcriptomics and metabolomics analyses demonstrated that some similar immune processes, including cytokine–cytokine receptor interaction, TNF signaling, and IL-6 production, and metabolic pathways were disturbed in SARS-CoV-2-infected mice as those observed in patients and organoid model^15,18,23,52^. Importantly, NAD^+^ system was coming under attack in the SARS-CoV-2 infection (as shown by Heer et al.^37^), and the supplementation of NAD^+^ and its intermediate, NMN, can effectively protect the lungs from the pathological damage and even death (by NMN). Besides, we found that two CD200 receptor genes, *Cd200r3* and *Cd200r4*, were upregulated in the lungs of SARS-CoV-2-infected mice. The expression level of *Cd200r3* coincided with the activation of macrophage and inflammatory injury^19,20^. CD200R3 and CD200R4 could interact with the activatory adapter protein, DAP12, and transmit strong activating signals^19,20^. CD200R3 can also function as an activating receptor in mast cells and basophils and plays a role in IgE-independent immune responses, and depletion of basophils with CD200R3 monoclonal antibody ameliorated IgE-mediated systemic and local anaphylaxis^53,54^. Therefore, *Cd200r3* may serve as a potential therapeutic target for the immune storm induced by SARS-CoV-2 infection. Interestingly, we found that NAD^+^ supplementation could effectively suppress *Cd200r3* expression, while NMN supplementation could effectively suppress the expression of both *Cd200r4* and *Cd200r3*. NAD^+^ intermediate and precursors, including NMN, NR, and NAM, have been shown to have anti-inflammatory effects in different animal models including aging, autoimmune encephalomyelitis, ischemia, Ataxia Telangiectasia and ZIKV infection^2,3,55–58^. Our results would provide more insights into the underlying mechanism.

NADPH oxidases are responsible for catalyzing the production of ROS which is important for the functions of phagosome during immune response^59^. As the expressions of NADPH oxidase genes, including *Cybb*, *Ncf2*, *Ncf4*, and *Rac2*, were significantly upregulated, NADPH would be over-consumed in the lungs of SARS-CoV-2-infected mice. NADPH can provide the reducing equivalents for biosynthetic reactions and the oxidation-reduction, and allow the regeneration of glutathione (GSH)^60^. Lower levels of NADPH can weaken the red blood cell membrane and trigger hemolysis as the maintenance of red blood cells require large amounts of GSH^61^. The hemolysis detected in the lungs and the upregulation of genes involved in the production of NADPH and NADP^+^, including *G6pdx*, *Pgd*, and *Nadk*, suggest the occurrence of NADPH deficiency and compensative effects through the increased expression of genes involved in NADPH synthesis in the infected lungs. Our study supports the notion that oxidative stress generated from ROS occurs in SARS-CoV-2-induced pneumonia^62^.

Consistent with our study, previous metabolomics studies showed that the level of tryptophan was decreased while the level of kynurenine was increased in the sera from severe COVID-19 patients^47,51,63^. The kynurenine/tryptophan ratio was higher in severe patients than in mild patients^51^, suggesting higher IDO activity in severe patients. Indeed, our transcriptomics data showed significantly higher expression level of *Ido1* in MASCp36-infected lungs than controls. Although high kynurenine/tryptophan was postulated to correlate with the severity of COVID-19 patients^51^, kynurenine can inhibit the activities of pathogenic CD4^+^ T cells^64^. Our results showed that the levels of both tryptophan and kynurenine were elevated by NMN supplementation, which implicated that the causal relationship between the level of kynurenine and the severity of COVID-19 is uncertain.

Our transcriptomics and qPCR results indicated that a set of PARP family members, including *Parp9*, *Parp10*, *Parp12*, and *Parp14*, were significantly induced by SARS-CoV-2 infection in mice, similar to those in COVID-19 patients^37^. PARPs can cleave NAD^+^ and catalyze poly-ADP-ribosylation or mono-ADP-ribosylation on target proteins, which play important roles in DNA damage repair. However, Nsp3 of SARS-CoV-2 was found to reverse the PARP9-mediated ADP-ribosylation recently^65^, which may in turn promote PARP activity and increase the demand for NAD^+^. However, our absolute quantification results showed that the levels of NAD^+^ precursors, NAM and NA, but not NAD^+^, NMN, or NR, were significantly downregulated after MASCp36 infection. In addition, the correlation between the decreased NMN levels in patients’ sera and the severity of COVID-19 was reported during the revision of this manuscript^50^. These results suggest that when the demand for NAD^+^ increases, NAM and NA were the first two NAD^+^ precursors to be consumed, followed by NMN and NR. Our absolute quantification results confirmed that the levels of NAD^+^, NMN, NR, and NAM were increased by NMN supplementation. Moreover, NMN supplementation significantly rescued many metabolites that were significantly reduced after SARS-CoV-2 infection, and helped to maintain the metabolic balance of SARS-CoV-2-infected lungs and protect the mice from death.

In this study, two mouse infection models were adopted. MASCp6-infected mice did not show very significant clinical symptoms that are present in COVID-19 patients, although pathological damages were detected in the lungs^13^. However, MASCp36 is the lethal mouse-adapted virus, and infected mice exhibit age- and gender-related skewed distribution of mortality akin to severe COVID-19^12^. Therefore, we used it to validate the therapeutic effects of NAD^+^ and NMN. Although our preliminary results were in support of clinical trials at the moment, there are fewer data about the dosage and long-term efficacy of NAD^+^ or NMN as a therapeutic drug in human studies at present. In a patent application, NMN was proposed as an anti-aging drug or healthcare supplement at doses of 1–500 mg/kg/day^41^. Further trials with different dosages and other clinical indicators remain to be evaluated for optimal therapeutic effect.

Taken together, based on our finding that NAD^+^ and NMN can alleviate the pathological damage of lungs and even death of SARS-CoV-2-infected mice and other groups’ studies of NAD^+^-boosting therapy for different diseases in humans^2,4^, trials for treating COVID-19 patients with NAD^+^, its intermediates or precursors should be considered.

## Materials and methods

### Ethics statement

All procedures involving animals were conducted in temperature- and humidity-controlled Biosafety Level 3 laboratory (BSL-3) and approved by the Animal Experiment Committee of Laboratory Animal Center, Beijing Institute of Microbiology and Epidemiology (approval number: IACUC-DWZX-2020-002). All animal experiments were handled in accordance with the recommendations in the Guide for the Care and Use of Laboratory Animals.

### Mice

All BALB/c mice were purchased from Bejing Vital River Laboratory Animal Technology Co., Ltd. Six- to seven-week-old and 8–9-month-old female mice were used for MASCp6 infection experiments and 8–9-month-old male mice were used for MASCp36 infection experiments.

### Virus strains

SARS-CoV-2 strains MASCp6 and MASCp36 were prepared as previously described^12,13^. All experiments involving infectious SARS-CoV-2 were performed in BSL-3 containment laboratory in Beijing Institute of Microbiology and Epidemiology.

### Mouse procedures

For MASCp6-infected mouse model, anesthetized 6–7-week- and 8–9-month-old female BALB/c mice were i.n. treated with a dose of 6 × 10^3^ plaque-forming unit (PFU) of MASCp6 in a total volume of 30 μL in each group. For MASCp36-infected mouse model, anesthetized 8–9-month-old male BALB/c mice were i.n. treated with a dose of 1200 PFU of MASCp36 in a total volume of 30 μL in each group.

The infected mice were i.p. administered with either NAD^+^ (or NMN) dissolved in 0.9% saline or the equivalent volume of 0.9% saline once a day for 3 consecutive days starting at half a day after inoculation of the virus. The dosages of NAD^+^ (Sigma, Cat# N7004) and NMN (EffePharm) are 1 mg/g/day and 0.5 mg/g/day, respectively. The mice were euthanized on 3 dpi by isoflurane overdose, and tissue samples were collected for subsequent experiments.

For survival experiment, 8–9-month-old male BALB/c mice were i.n. treated with a dose of 300 PFU for survival experiment of MASCp36 in a total volume of 30 μL in each group. The infected mice were i.p. administered with NMN dissolved in 0.9% saline or the equivalent volume of 0.9% saline once a day until the end of the experiment starting at half a day after inoculation of the virus. The dosage of NMN is 0.5 mg/g/day.

### Sample preparation

For histopathological and immunofluorescent staining, the tissues of each mouse were removed and fixed in 4% paraformaldehyde at 4 °C for 2 days. For RNA extraction, the tissues were homogenized and lysed with TRIzol reagent (Life Technologies, 15596018), and RNA was extracted according to the manufacturer’s instructions. For metabolomics, the tissues were weighed and homogenized with 75% ethanol (1:50, tissue weight (mg):volume (μL) of 75% ethanol). The same lobe of the lung from each mouse was collected for the same analysis.

### Measurement of viral RNA

Viral RNA (vRNA) was extracted using the QIAamp Viral RNA Mini Kit (Qiagen, Cat# 52906) according to the manufacturer’s protocol. vRNA quantification in each sample was performed by qPCR targeting the S gene of SARS-CoV-2 as described previously^13^.

### Transcriptomics

Total RNAs of lungs from MASCp6-infected 8–9-month-old BALB/c mice and control mice were sent to LC-Bio Technology (Hangzhou) for sequencing and analysis. Purified RNA was subjected to the 2× 150 bp paired-end sequencing (PE150) on an Illumina Novaseq™ 6000 (LC-Bio Technology Co., Ltd., Hangzhou, China) following the vendor’s recommended protocol. After preprocessing, the high-quality reads were compared with the mouse reference genome (ftp://ftp.ensembl.org/pub/release-99/fasta/mus_musculus/dna/) by HISAT2 software (https://daehwankimlab.github.io/hisat2/).

The significantly differentially expressed mRNAs were selected with |Log_2_FC| > 1 and *P* value < 0.05 by R package DESeq2^66^. GO enrichment analysis and KEGG enrichment analysis of significantly differently expressed genes were performed using the OmicStudio tools at https://www.omicstudio.cn/tool. Gene number and *P* value of GO and KEGG terms were visualized using the R package ggplot2. The network of the genes among different GO and KEGG terms were visualized using Cytoscape (version 3.7.2). The heatmaps and bar plots of significantly differently expressed genes were drawn with the R package ggplot2 and GraphPad (Prism 7), respectively.

### RT-qPCR

1.5 μg of total RNA was used for RT-qPCR using Reverse Transcription Kit (Promega, A3500) according to the manufacturer’s protocol. Then, cDNA was diluted by 1:4 and 1 μL diluted cDNA was used for qPCR with qPCR mix (Bio-Rad, Cat# 1725201). qPCR was performed on Bio-Rad CRX96 Real-Time PCR system.

### Histopathological staining and analysis

Four percent paraformaldehyde-fixed lung tissues for histological examination were subjected to alcohol gradient dehydration and embedded in paraffin. Each embedded tissue was sectioned at 5 μm thickness sections. At least three sections of each tissue were used for staining with H&E (Solarbio, Cat# G1120) and examined under a light microscopy. The tissue handling and H&E staining followed standard protocol.

For quantification of inflammatory cell aggregation number and area and alveolar septum relative area, inflammatory cell aggregation was defined by dense cell cluster and dark blue staining with hematoxylin, and the relative area of alveolar septum was calculated as the total area of the substances divided by the area of whole lung section.

### Immunofluorescent staining and analysis

Four percent paraformaldehyde-fixed lung tissues were dehydrated in 30% sucrose for 2 days and embedded in optimal cutting temperature compound (O.C.T. compound, SAKURA, 4583, USA). 30 μm sections were sliced with freezing microtome (CM 1950, Leica, Germany). Sections were blocked with blocking buffer (PBS + 10% FBS + 3% BSA + 0.2% Triton X-100) at room temperature for 1 h and incubated with the primary antibodies at 37 °C for 2 h. After washing with PBST (PBS + 0.2% Triton X-100) for 3 times (10 min for each time), the lung sections were subsequently incubated with fluorescent secondary antibodies at room temperature for 1 h, followed by washing with PBST for 3 times (10 min for each time). Primary antibodies include: rabbit anti-cleaved caspase 3 (1:500, Cell Signaling Technology, Cat# 9664), rabbit anti-CD68 (1:1000, Abcam, Cat# ab125212), rabbit anti-SARS-CoV-19 nucleocapsid (1:100, Sino Biological, Cat# 40588-T62), human/mouse anti-SARS-CoV-19 spike (1:100, Sino Biological, Cat# 40150-D001). Secondary antibodies were Alexa Fluor 488-, Alexa Fluor 568-, and Alexa Fluor 647-conjugated goat secondary antibodies against rabbit and human IgG (1:2000; Invitrogen). Nuclei were counterstained with DAPI (Cell Signaling Technology, Cat# 4083s). Fluorescent images were captured using a confocal laser-scanning microscope (Carl Zeiss, LSM700 and LSM980) and analyzed with ZEN3.1 and ImageJ.

For quantification of blocked airway ratio and relative area, blocked airway ratio was calculated as the number of airways having blockage divided by the number of total airways in each section, and the relative area of blockage was calculated as the area of each embolism divided by the area of the corresponding airway. For quantification of the ratio of caspase 3^+^ cells, the ratio of caspase 3^+^ cells was calculated as the number of caspase 3^+^ cells divided by the number of infected cells in the same location of the adjacent section. For quantification of SARS-CoV-2 relative fluorescence intensity and infected area, relative fluorescence intensity was calculated as the total fluorescence intensity of SARS-CoV-2 signal divided by the area of whole lung section. The relative infected area was calculated as the area of minimal convex polygon which can cover all of virus signal divided as the area of whole lung section.

### Metabolite extraction and metabolomics

Homogenate of lung tissues was vortexed for 1 min. Then, 100 μL of homogenate was transferred into a tube pre-added with 900 μL of ice-cold 80% methanol, vortexed for 1 min and centrifuged at 14,000 rpm, 4 °C for 15 min. 900 μL of supernatant was collected into a new tube. 500 μL of 80% methanol was added to the pellet, vortexed for 1 min, and centrifuged at 14,000 rpm, 4 °C for 15 min. 500 μL of supernatant was collected and mixed with 900 μL of supernatant collected previously. Supernatant was evaporated to dryness under a speed vacuum concentrator. Dried metabolite pellets were kept at −80 °C until analysis.

Dried metabolite pellets were dissolved in 0.3% formic acid in water. Chromatographic separation was performed on a Nexera UHPLC system (Shimadzu), with a RP-UPLC column (HSS T3, 2.1 mm × 150 mm, 1.8 μm, Waters) and the following gradient: 0–3 min 99% A; 3–15 min 99%–1% A; 15–17 min 1% A; 17–17.1 min 1%–99% A; 17.1–20 min 99% A. Mobile phase A was 0.03% formic acid in water. Mobile phase B was 0.03% formic acid in acetonitrile. The flow rate was 0.25 mL/min, the column was at 35 °C and the autosampler was at 4 °C. Mass data acquisition was performed using an AB QTRAP 6500+ triple quadrupole mass spectrometer (SCIEX, Framingham, MA) in multiple reaction monitoring (MRM) mode for the detection of 260 unique endogenous metabolites as previously described^11,50,67,68^. Chromatogram review and peak area integration were performed using MultiQuant software v.3.0.2 (SCIEX). Raw peak areas were used as variables for multivariate and univariate statistical data analysis.

Metabolites with significant differences (*P* < 0.05 and fold change < 0.8 or > 1.25) were used for KEGG enrichment analysis using MetaboAnalyst 5.0 (https://www.metaboanalyst.ca/). Metabolic pathways with FDR < 0.05 were considered significant.

## Supplementary information

Sumpplementary Figures

Sumpplementary Table S1

Sumpplementary Table S2

## Data Availability

The RNA-seq data generated in this study have been deposited in Genome Sequence Archive (BSA) database (https://ngdc.cncb.ac.cn/gsa/) under accession code CRA003464. Metabolomics data are included in Supplementary Tables S1 and S2.
