# Supplementary material for: Treatment of SARS-CoV-2-induced pneumonia with NAD+ and NMN in two mouse models
Source: Cell Discov. 2022 Apr 29;8:38. doi: 10.1038/s41421-022-00409-y (PMC9053567; doi:10.1038/s41421-022-00409-y)
Supplement: Supplementary file 1 — Sumpplementary Figures [file 41421_2022_409_MOESM1_ESM.pdf]

Supplementary Figure 1

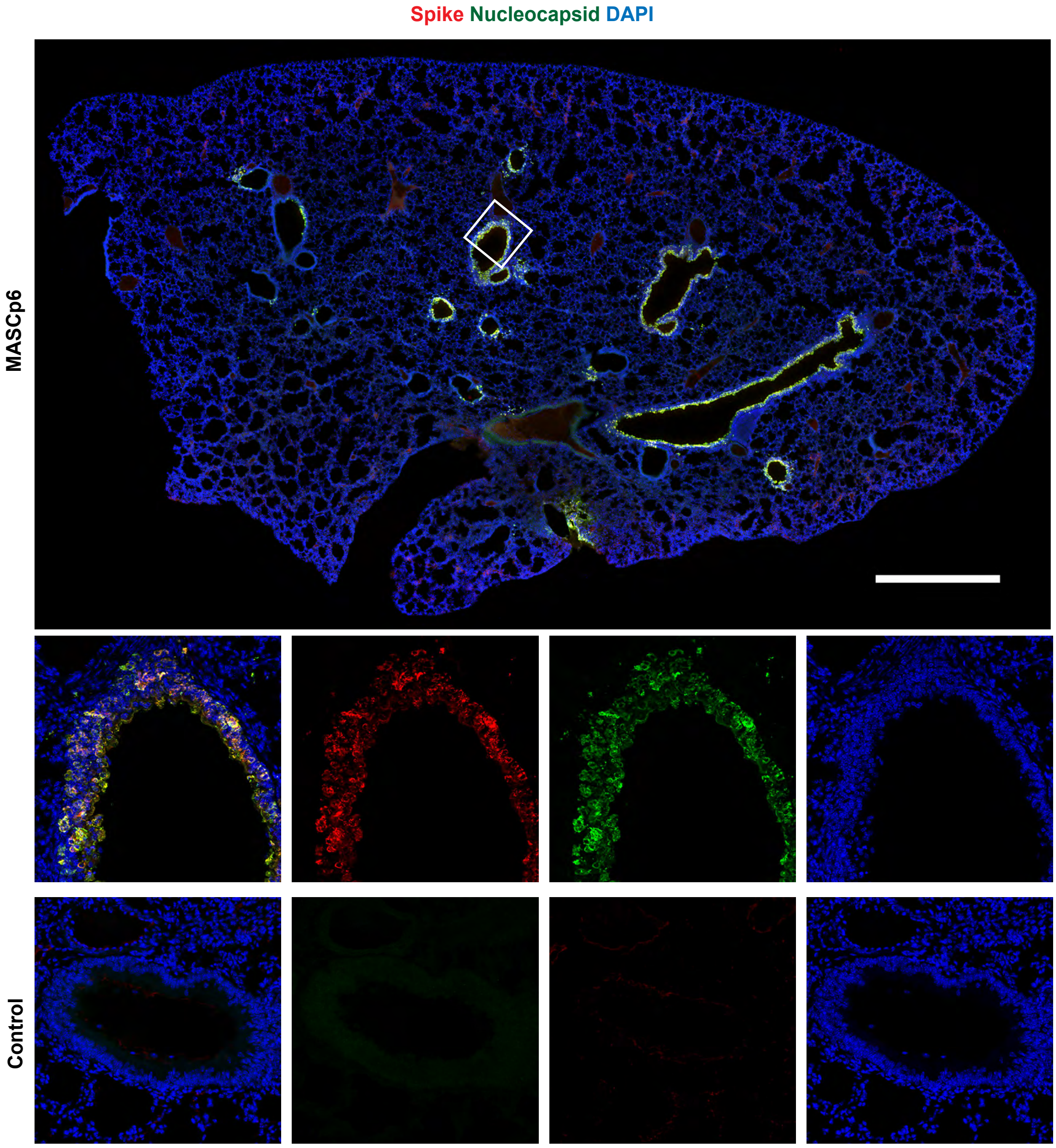

Supplementary Fig. S1 Infection of mouse-adapted SARS-CoV-2 strain MASCp6 in trachea epithelial cells. Immunostaining results of SARS-CoV-2 spike protein (red) and nucleocapsid protein (green) and DAPI (blue) in the lung sections of SARS-CoV-2 infected mice and control mice.

Supplementary Figure 2

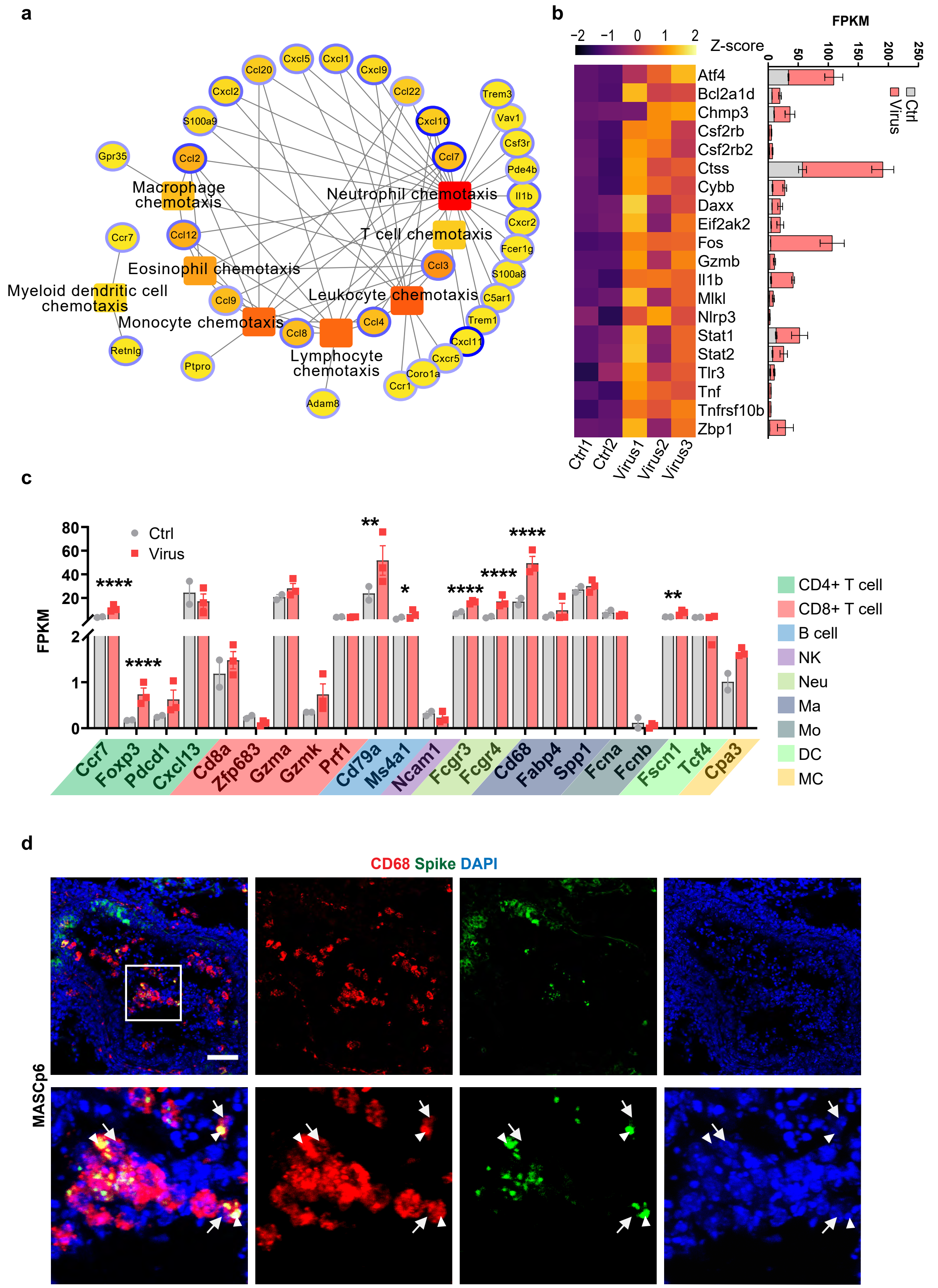

Supplementary Fig. S2 Immune response of MASCp6-infected mouse lungs. a Network of chemotaxis related genes grouped by GO terms of significantly upregulated genes. The fill color represents the number of related nodes: yellow (low) to red (high). The border color represents the fold change: light blue (low) to deep blue (high). **b** Heat map and bar plot show relative expression levels of significantly differently expressed genes involved in necroptosis and apoptosis. **c** Bar plot shows relative expression levels of some marker genes of immune cells. NK: natural killer cell; Neu: neutrophil; Ma: macrophage; Mo: monocyte; DC: dendritic cell; MC: mast cell. **d** Immunostaining results with SARS-CoV-2 spike (green) and macrophage marker (CD68, red) antibodies show virus infected cells were phagocytized by macrophage. Arrow: macrophages; Arrow head: infected cells. Scale bars, 50 μm. All gene expression data are from RNAseq. All data showed as means ± s.e.m. *p < 0.05, **p < 0.01, ***p < 0.001, ****p < 0.0001.

Supplementary Figure 3

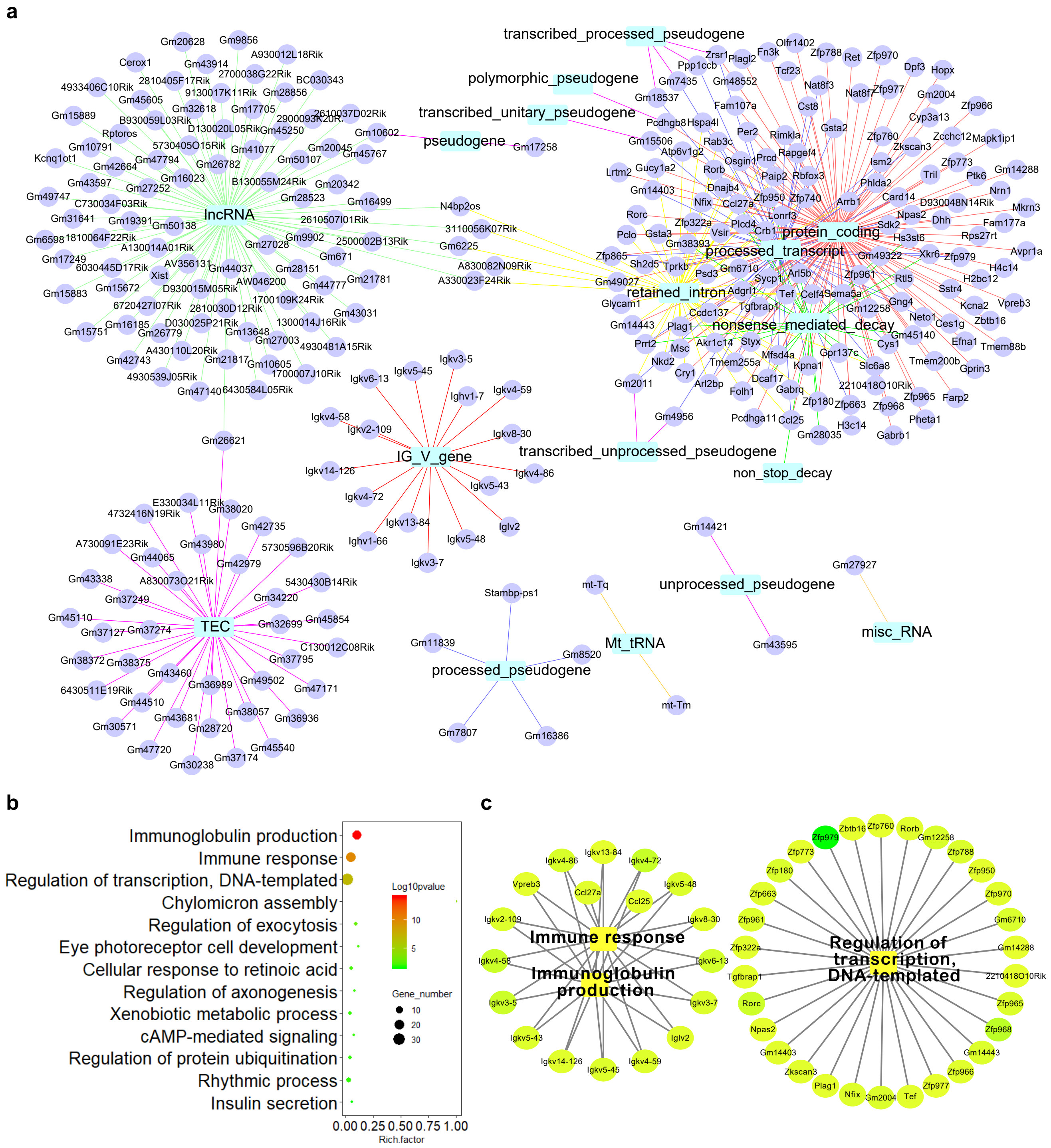

Supplementary Fig. S3 Significantly downregulated genes of MASCp6-infected mouse lungs detected by RNAseq. a Network of significantly downregulated genes grouped by gene biotype. **b** GO analysis of significantly downregulated genes. Rich factor represents the ratio of significantly differently expressed genes to the total genes belonged to the GO term. **c** Network of genes belonging to top3-enriched GO terms of significantly downregulated genes. The fill color represents the fold change: yellow (low) to green (high).

Supplementary Figure 4

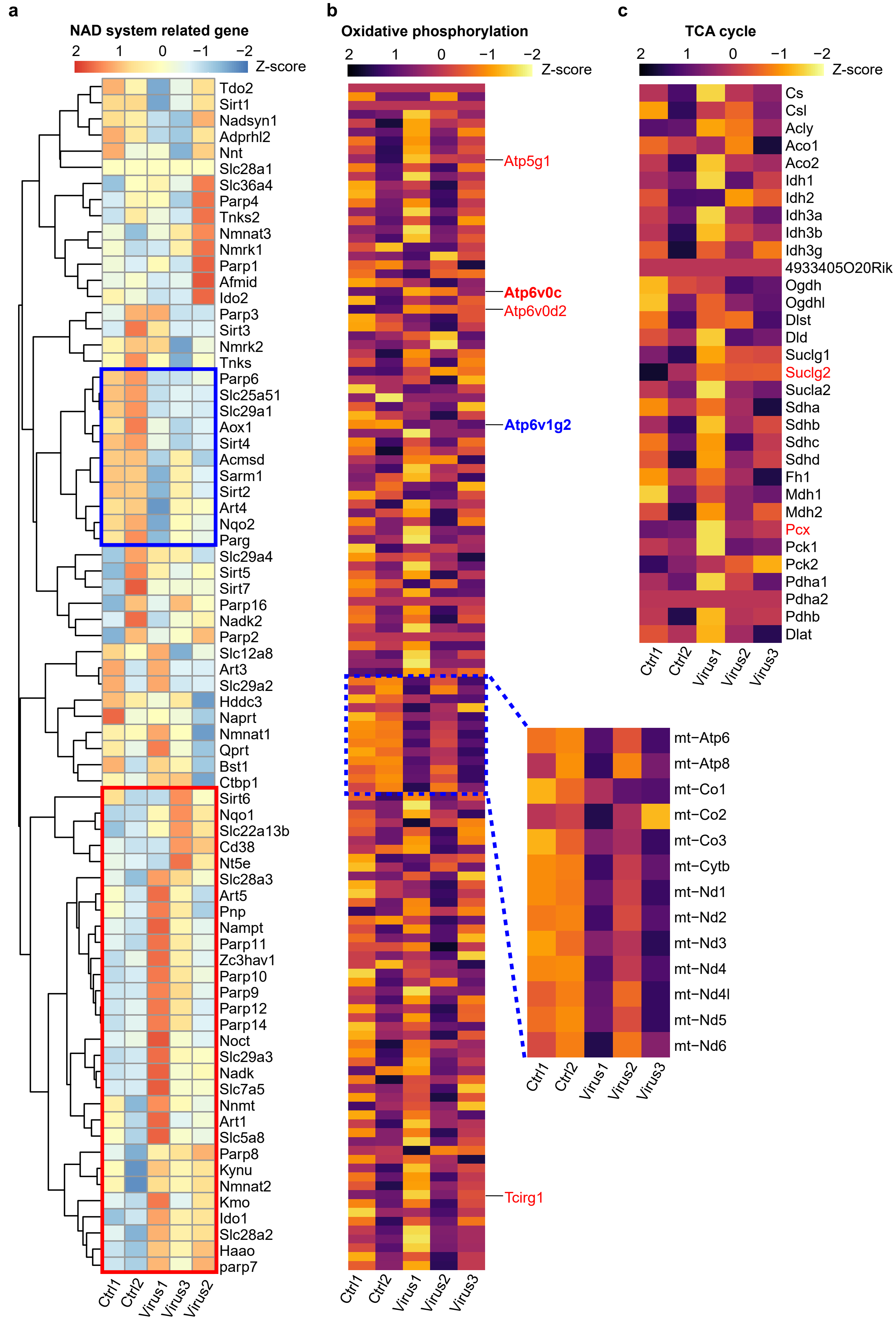

Supplementary Fig. S4 Overview of relative expression levels of NAD^+ system and mitochondrion related genes detected by RNAseq in MASCp6-infected mice and control mice. a Heat map shows relative expression levels of 74 genes belonging to NAD^+ system (Refer to Heer et al., 2020). Red border: genes significantly upregulated or tended to upregulated by MASCp6 infection. Blue border: genes significantly downregulated or tended to downregulated by MASCp6 infection. **b**,**c** Heat maps show relative expression levels of oxidative phosphorylation (**b**) and TCA cycle (**c**) related genes. Blue dotted box in (**b**): genes encoded by mitochondrial DNA. Red gene: upregulated by MASCp6 infection. Blue gene: downregulated by MASCp6 infection.

Supplementary Figure 5

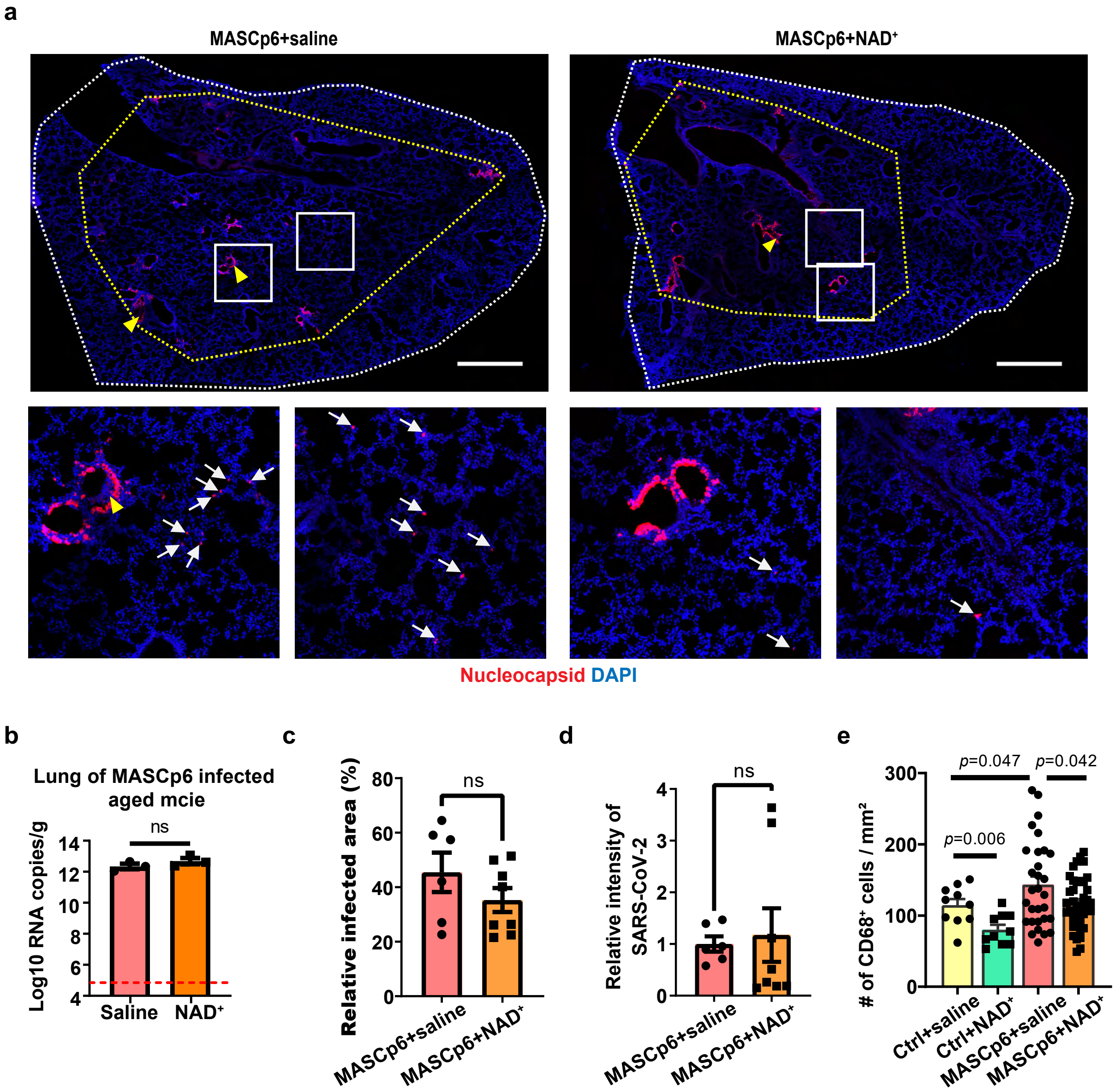

Supplementary Fig. S5 NAD$^+$ supplementation has no effect on multiplication of MASCp6 in mice. a Immunostaining results of SARS-CoV-2 nucleocapsid protein (red) and DAPI (blue) of lung sections from saline and NAD$^+$ administrated MASCp6-infected mice. Yellow arrow head: airway blockage; White arrow: virus infected alveolar epithelial cells; Yellow frame: virus infected area; white frame: total area. Scale bars, 1000 μm. **b** Bar plot indicates the expression levels of viral RNA detected by qPCR in lungs of saline and NAD$^+$ administrated MASCp6-infected aged mice (8-9 months old). n = 3 mice per group. **c**,**d** Quantification of the relative fluorescence intensity (**c**) and infected area (**d**) of MASCp6. n = 3 mice for MASCp6 + saline group, n = 4 mice for MASCp6 + NAD$^+$ group. **e** Quantification of the density of CD68 positive cells of lung sections. n = 2 mice for Ctrl + saline group and Ctrl + NAD$^+$ group, n = 3 mice for MASCp6 + saline group, n = 4 mice for MASCp6 + NAD$^+$ group. All quantification data showed as means ± s.e.m, t test. Exact p values are indicated.

Supplementary Figure 6

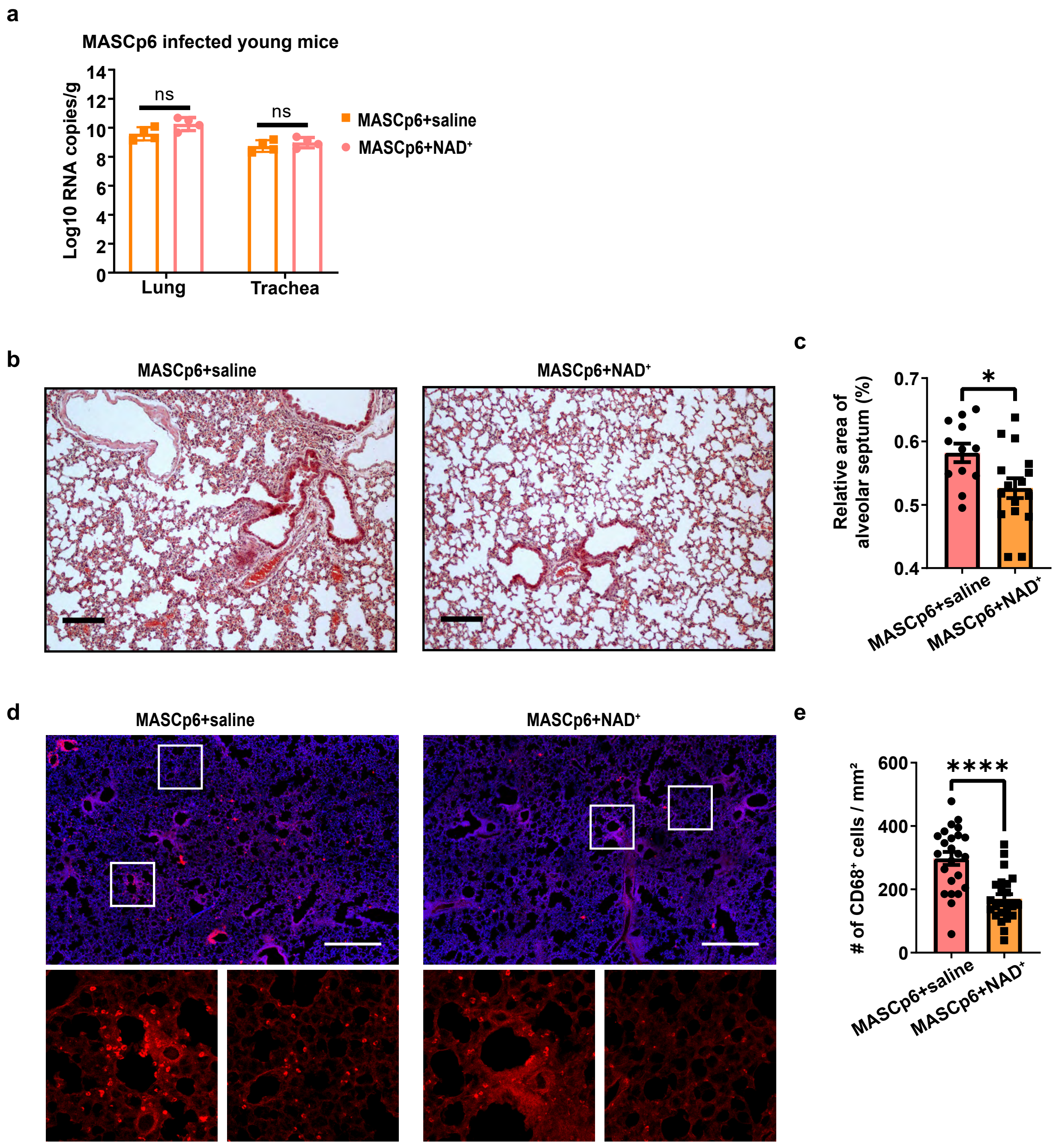

Supplementary Fig. S6 Protective effect of NAD^+ supplementation for pathological changes caused MASCp6 infection in young mice (6-7 weeks old). a Bar plot indicates the expression levels of viral RNA detected by qPCR in lungs and tracheas of saline and NAD^+ administrated MASCp6-infected mice respectively. **b** H&E staining results show alveolar septal thickening was rescued by NAD^+ supplementation in lung sections of saline and NAD^+ administrated MASCp6-infected mice. Scale bars, 200 μm. **c** Quantification of the relative area of alveolar septum in lung sections from saline and NAD^+ administrated MASCp6-infected mice. **d** Immunostaining results with macrophage marker (CD68, red) antibody and DAPI (blue) show macrophage infiltration was rescued by NAD^+ supplementation in lung sections of saline and NAD^+ administrated MASCp6-infected mice. Scale bars, 500 μm. **e** Relative abundances of tryptophan, kynurenine, glucose, γ-aminobutyric acid and NAM in the NMN group and saline group of MASCp36-infected mice. Relative abundance of each metabolite in control mice was normalized to 1. All quantification data shown as mean ± s.e.m. t test. Exact p values are indicated. n = 3 mice for each group. **f** Quantification of the density of CD68 positive cells in lung sections of saline and NAD^+ administrated MASCp6-infected mice. All quantification data showed as means ± s.e.m, t test. *$p < 0.05$, ****$p < 0.0001$. n = 4 mice for each group.

Supplementary Figure 7

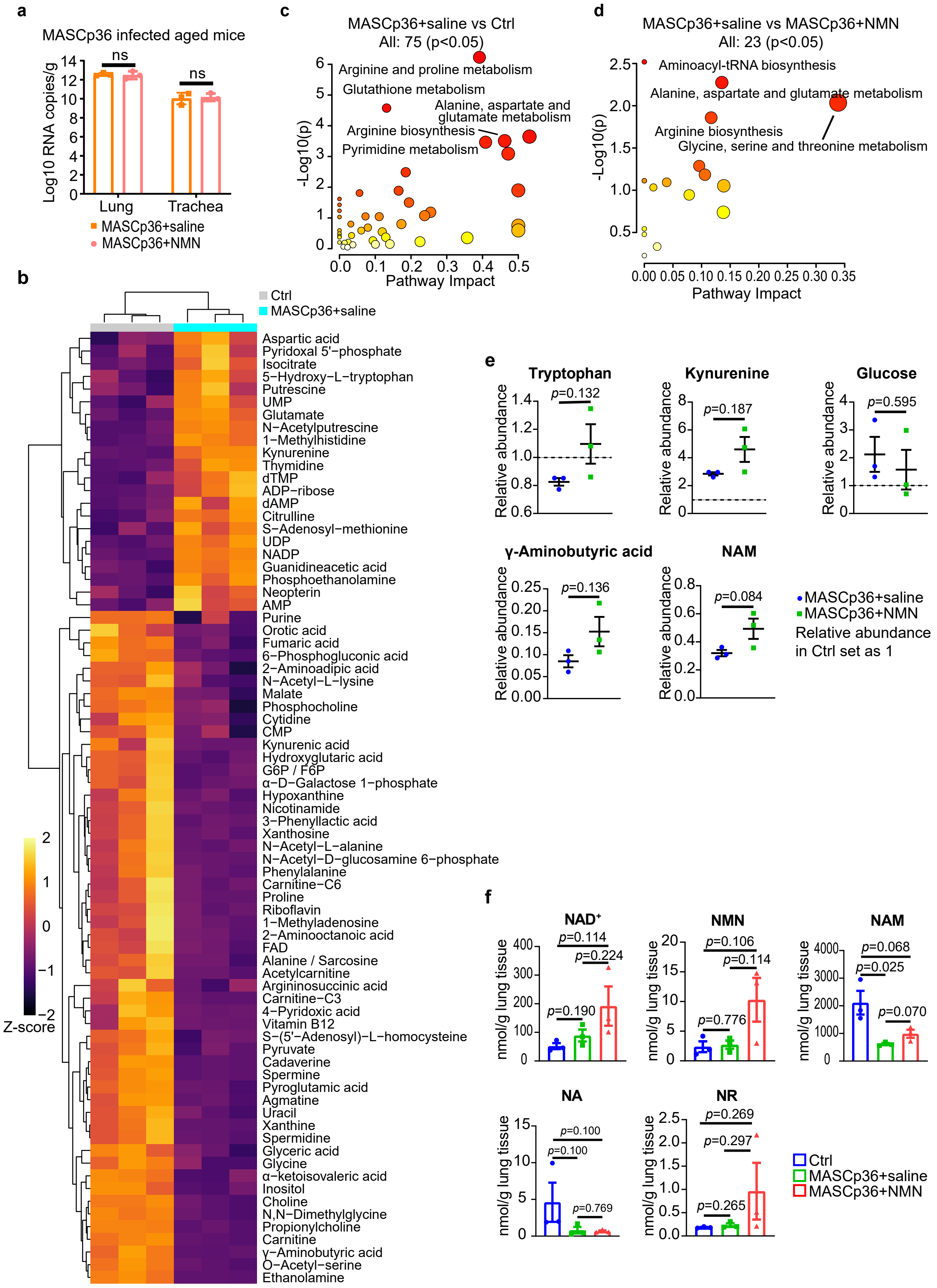

Supplementary Figure 7. Treatment of MASCp36 induced pneumonia with NMN and NAD^+ in aged mice (8-9 months old). a Bar plot indicates the expression levels of viral RNA detected by qPCR in the lungs of saline, NMN administrated MASCp36-infected aged mice (8-9 months old). **b** Heat map shows relative abundance of significantly differential metabolites ($p < 0.05$; $FC < 0.8$ or $FC > 1.25$) in the saline group of MASCp36-infected mice compared with control mice. **c** KEGG analysis of significantly differential metabolites in the saline group of MASCp36-infected mice compared with control mice. **d** KEGG analysis of significantly differential metabolites in the NMN group compared with saline group of MASCp36-infected mice. **e** Absolute quantification of NAD^+, NMN, NR, NAM and NA in the NMN group and saline group of MASCp36-infected mice and control mice. All quantification data showed as means ± s.e.m. Mann Whitney test for NA, and t test for others. n = 3 mice for each group. Exact p values are indicated.
